# Exploring the role of systemic inflammation in guiding clinical decision making for geriatric patients with a hip fracture

**DOI:** 10.1007/s00068-025-02875-x

**Published:** 2025-05-06

**Authors:** E. J. de Fraiture, T. M. P. Nijdam, F. J. C. van Eerten, H. J. Schuijt, A. Bikker, L. Koenderman, F. Hietbrink, D. van der Velde

**Affiliations:** 1 Department of Trauma Surgery, UMC Utrecht, Utrecht, Netherlands; 2 Department of Trauma Surgery, St. Antonius Hospital, Utrecht, Netherlands; 3 Department of Clinical Chemistry, St. Antonius Hospital, Utrecht, Netherlands; 4 Department of Respiratory Medicine, UMC Utrecht, Utrecht, Netherlands; 5 Center for Translational Immunology (CTI), UMC Utrecht, Utrecht, Netherlands

**Keywords:** Geriatric, Hip fracture, Inflammation, Neutrophil, Trauma, Flow cytometry

## Abstract

**Purpose:**

Geriatric patients with a hip fracture are at risk for adverse outcomes after surgery. A pilot study showed the feasibility of assessing of systemic inflammation in these patients through neutrophil analysis. The aim of this study was to correlate neutrophil categories to clinical outcomes in a larger cohort.

**Methods:**

In this prospective cohort study, blood samples were taken from geriatric patients with a hip fracture directly after trauma and healthy older people serving as controls. Neutrophil phenotypes were categorized (0–6 from no inflammation to severe inflammation) and correlated to clinical outcomes.

**Results:**

In total, 289 patients (median age 82) and 45 age matched controls were included. Severe infections occurred in 8% of the patients and 9% died within 30 days. Patients displayed all neutrophil categories (0–6), while controls showed categories 0,1,3. A newly identified neutrophil category had higher leukocyte counts and CRP, with trends toward increased infections and mortality. Among patients receiving palliative care, 30-day mortality was 50% in categories 0–1 and 83% in higher categories.

**Conclusion:**

Neutrophil categories offer a feasible method to assess systemic inflammation and may assist in shared decision-making for palliative care. The data are consistent with the hypothesis that patients in category 0–1 are deemed fit for surgery, when other risk factors are absent. However, further research should investigate the quality-of-life of patients still alive after 30 days in order to determine whether immune profiling is of added clinical value in decision making regarding traumatic hip fractures in geriatric patients.

**Supplementary Information:**

The online version contains supplementary material available at 10.1007/s00068-025-02875-x.

## Introduction

Geriatric patients with a hip fracture are a fast-growing and heterogeneous group, with high prevalence of frailty and a high risk of adverse outcomes after surgery [1–6]. While hip fractures are generally treated operatively [5, 7, 8], palliative non-operative management (NOM) can be a valuable alternative for geriatric patients with limited life expectancy and reduced quality of life [8–11]. This approach focuses on palliative care and pain relief instead of an attempt of restoring mobility and independence [12]. Recent studies have shown that NOM is comparable to operative management in terms of quality-of-life for geriatric patients with limited life expectancy [10]. Consequently, shared decision-making (SDM) has become essential in the acute setting to align treatment choices with patients' needs and wishes [9, 11].

The decision-making process can be challenging for clinicians, patients, and families, as most geriatric trauma patients have not previously considered palliative treatment options before their arrival at the emergency department (ED) [12]. Furthermore, due to time constraints regarding treatment decisions in the acute setting there is typically limited time for SDM [8, 13, 14]. A key challenge in this context is the difficulty in predicting outcomes for this patient population. Although many prediction models have been developed, none have demonstrated consistent reliability [15, 16]. In the absence of prior advance care planning, objective criteria that help identify patients at high risk for adverse outcomes could support these complex treatment decisions.

A recent pilot study demonstrated the feasibility of point-of-care (PoC) fully automated flow cytometry at the ED to analyze the neutrophil compartment in geriatric patients with a hip fracture [17]. This study showed that patients with a healthy neutrophil phenotype developed less severe adverse outcomes, suggesting that early changes in the neutrophil compartment may serve as diagnostic for determination whether patients are fit for surgery [17]. Upon validation, these objective immune markers could enhance SDM by providing measurable indicators of risk. This may support clinicians and patients in making well-informed treatment choices.

Recent advances in fully automated flow cytometry for clinical settings now allow for rapid, precise and 24/7 measurement of neutrophil categories without manual sample processing [18, 19]. Building on findings from the pilot study, this research hypothesized that early changes in neutrophil categories are associated with adverse outcomes, such as delirium, infectious complications, in-hospital mortality, and 30-day mortality. This study aimed to contribute to the identification of high-risk patients through objective cellular markers, thereby guiding personalized treatment strategies for this patient population.

## Methods

### Study design

A prospective cohort study in geriatric patients with a hip fracture, presented at the ED was conducted. Patients were included between December 2022 and July 2024. Patients were eligible for inclusion if they sustained a subtrochanteric, per trochanteric or femoral neck fracture < 24 h before presentation at the ED, were aged > 70 years and underwent diagnostic blood sampling as part of the standard-of-care treatment during acute trauma care. Patients were excluded if they suffered multi trauma (defined as Injury Severity Score ≥ 16) [20], if they were transferred from another hospital > 24 h after trauma, if they were transferred to another hospital after presentation at the ED or if they were immunocompromised.

The medical ethical committee MEC-U, Utrecht, The Netherlands, approved this study under protocol no. R22.067. The study was approved and registered by the Central Committee on Research Involving Human Subjects in the Netherlands under protocol no. NL81682.100.22 and was performed in accordance with the ethical standards established by the Declaration of Helsinki and its later amendments.

### Controls

Age matched volunteers were included in September 2023. All volunteers enlisted themselves via the St. Antonius Research Foundation. Inclusion criteria were age > 70 years and having either no comorbidities or chronic stable comorbidities that did not require acute medication or were managed with chronic medication. Participating age matched volunteers visited the outpatient clinic of the St. Antonius Hospital. During this visit, informed consent was obtained, and venipuncture was performed by drawing 4 mL of blood in a sodium heparin tube (Vacuette, Greiner Bio-One, Kremsmünster, Austria). The medical research ethics committee (MEC-U, Utrecht) approved this under protocol no. R.22.106, which is registered by the Central Committee on Research Involving Human Subjects in the Netherlands under protocol no. NL83286.100.22.

### Automated flow cytometry

From each geriatric patient with a hip fracture, blood was collected in a 4 mL sodium heparin tube (Vacuette; Greiner Bio-One) at the ED. Blood tubes were sent via pneumatic tube transport to the central laboratory and placed into the automated AQUIOS CL “Load & Go” flow cytometer (Beckman Coulter Life Sciences, Miami, FL, USA) within one hour after blood withdrawal. The impact of pneumatic tube transport on neutrophil activation was assessed by measuring duplicate healthy control blood samples, one transported via pneumatic tube and one without, in 8 healthy controls, with no differences observed. Data are shown in the Supplementary Information. The flow cytometer performs a fully automated sample preparation and flow cytometry analysis in whole blood samples, as earlier described in more detail [21]. In short, each sample was measured with a neutrophil antibody panel (CD16-FITC/clone 3G8, CD11b-PE/clone Bear1, CD62L-ECD/clone DREG56, CD10-PC5/clone ALB1, and CD64-PC7/clone 22). All antibodies were obtained from Beckman Coulter (Beckman Coulter Life Sciences, Miami, FL, USA). Identical sample analysis was performed in September 2023 with the blood of the age matched controls.

### Neutrophil phenotype categories

During acute inflammation, neutrophils can be divided into different subsets, based on the expression of specific surface proteins (CD16/FcγRIII and CD62L/L-selectin) [22]. A recent study defined neutrophil phenotype categories (0–6), based on the visual recognition of the distribution of neutrophil subsets, which are valuable for interpretation of the inflammatory response to trauma [19]. Category 0 displays a normal, healthy, homogenous neutrophil phenotype, while categories 1–6 deviate in an ascending order from normal. Category 1 only shows a (larger) CD62L^low^ subset, and Category 2 consists of one mixed subset in the lower left quadrant of the dot plot, where the expression of both CD16 and CD62L tends to be low. Category 3 consists of two additional small CD16^low^ and CD62L^low^ subsets which are similar in size, Category 4 consists of bigger CD16^low^ and CD62L^low^ subsets, and Category 5 shows extensive CD16^low^ and CD62L^low^ subsets, where the CD16^low^ subset is bigger than the CD62L^low^ subset. Category 6 shows an immunophenotype of mainly neutrophil progenitors, as well as CD62L^low^ neutrophils (the different categories are visualized in Table 3).

All patients and healthy controls were subdivided into one of the neutrophil phenotype categories. Categorization was done by visual assessment by two ‘blinded’ researchers as no existing algorithm can do this unbiased (not shown).

### Clinical data

All clinical data were extracted from the electronic patient file by the researcher (EF). Study data were collected and managed using REDCap (Research Electronic Data Capture) electronic data capture tools hosted at the St. Antonius hospital [23, 24]. REDCap is a secure web-based software platform designed to support data capture for research studies. The following demographic data and clinical parameters were collected: age, sex, Charlson Comorbidity Index (CCI) [25], ASA Physical Status Classification (I to V) [26], living situation (home, home with ADL care, nursing home), infections on admission, trauma mechanism, type of hip fracture, type of treatment, diagnosis of delirium, infectious complications, length of hospital stay, in hospital mortality and 30-day mortality. Severe infectious complications were defined as sepsis and/or pneumonia. The collected laboratory parameters were serum albumin, C-Reactive Protein (CRP) and leukocyte count at presentation,

### Statistical analysis

All data were analyzed in R Studio Version 4.4.1 [27]. Data are presented as median with interquartile range. Groups were compared using Mann–Whitney-Wilcoxon tests. A *p* value of < 0.05 between groups was considered as a statistically significant difference.

## Results

**Fig. 1:**
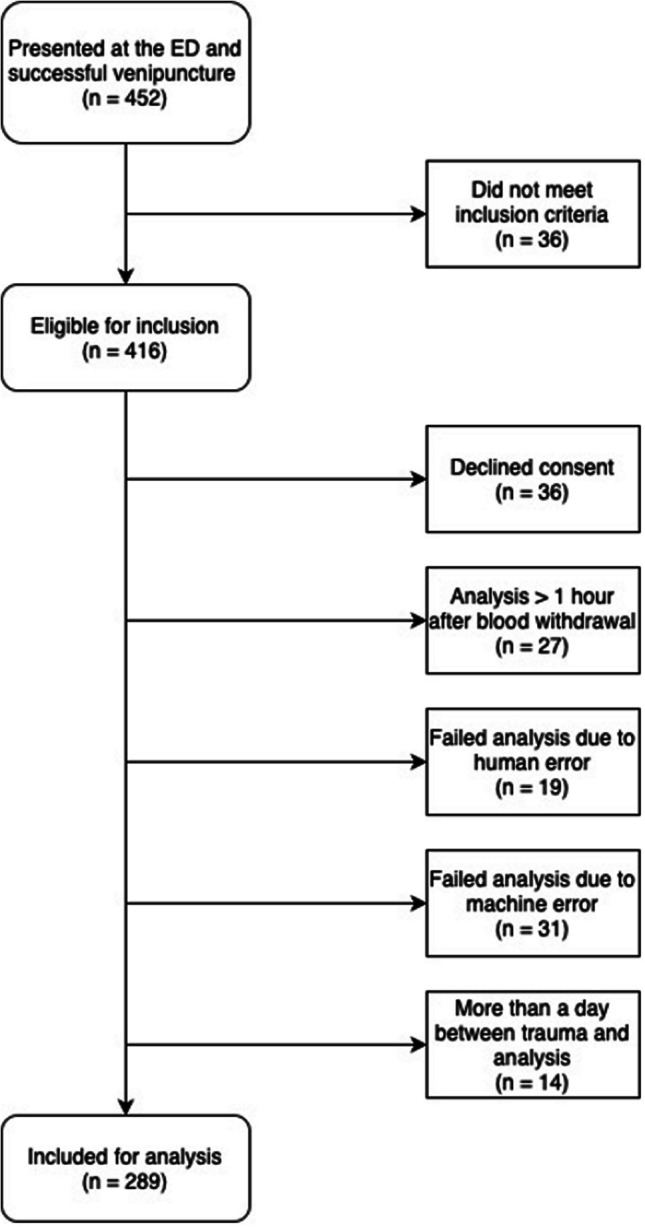
Flowchart of patient inclusion

Between December 2022 and July 2024, 452 patients with hip fractures presented to the ED and underwent a diagnostic workup. Among these, 36 patients (8%) did not meet the inclusion criteria, leaving 416 eligible patients. Consent was declined by 36 patients (9%), and analysis was unsuccessful in 91 patients (22%) due to one or more of the following reasons: over one hour between blood withdrawal and analysis, more than one day between trauma and blood sampling, or technical issues related to machine or human error. Consequently, 289 patients were included in the final analysis (Fig. 1).

### Baseline patient characteristics

The study included 289 patients, with a median age of 82 years (range 70–98), and predominantly female (64%). At admission, 14% of the patients presented with an infection, primarily urinary tract infections (85%). The presence of comorbidity was high, as indicated by ASA classifications: most patients were classified as ASA 2 (43%) or ASA 3 (49%), with a median Charlson Comorbidity Index (CCI) of 5 (range 3–11). The majority of patients lived independently at home (73%), while 13% received ADL care at home and 12% resided in nursing homes. Regarding injury and treatment, 91% of fractures were due to low-energy falls. Fracture types mainly included displaced femoral neck fractures (52%) and pertrochanteric fractures (35%). Surgical interventions varied, with 44% receiving hemi-arthroplasty and 36% intramedullary osteosynthesis. Palliative treatment was chosen for 30 (10%) patients of whom 21 (70%) died within 30-days. Median Almelo Hip Fracture Score (AHFS) [15] of patients receiving palliative care was 12 (range 8–18) (Table 1).

**Table 1:** Patient characteristics

Patient Characteristics	*N* = 289
Age (years)	82 [77–87] (70–98)
Sex female	185 (64)
Hospital admission	286 (99)
Infection on admission	41 (14)
* Urinary tract infection*	35 (12)
* Pneumonia*	4 (1)
* COVID 19*	4 (1)
* other*	2 (0.7)
ASA	
*1*	6 (2)
*2*	123 (43)
*3*	142 (49)
*4*	18 (6)
CCI	5 [4–6] (3–11)
Origin	
*Home*	212 (73)
*Home with ADL care*	39 (13)
*Nursing home*	34 (12)
*Other*	4 (1)
NMS	7 [5–9] (0–9)
BMI (kg/m^2)	25 [22–27] (13–41)
Leukocyte count (10^9/L)	10 [8–13] (4–24)
CRP (mg/L)	2 [1–9] (0–163)
Albumin (g/L)	41 [39–43] (28–48)
Mechanism of injury	
*Low energy fall*	264 (91)
*Fall from height*	8 (3)
*Traffic accident*	15 (5)
*Other*	2 (1)
Type of fracture	
*Femoral neck (displaced)*	148 (52)
*Femoral neck (undisplaced)*	23 (8)
*Pertrochanteric*	101 (35)
*Other*	17 (6)
Procedure	
*Dynamic hip screw*	18 (7)
*Cannulated screws*	5 (2)
*Hemi-arthroplasty*	114 (44)
*Total hip arthroplasty*	22 (9)
*Intramedullary osteosynthesis*	92 (36)
*Other*	7 (3)
Palliative treatment	30 (10)
*AHFS*	12 [11–14] (8–18)
*Deceased* < *30 days*	21 (7)

Data depicted as median [IQR] (min–max), or as absolute count (percentage), *CCI* = Charlson Comorbidity Score, *ADL* = activities of daily living, *NMS* = New Mobility Score, *BMI* = body mass index, *CRP* = C-reactive protein, *AHFS* = Almelo Hip Fracture Score

### Clinical outcome

The median hospital length of stay was 7 days (range 1–49). In total, 18% of the patients suffered from infectious complications after admission, with urinary tract infections (31%) and pneumonia (37%) most frequently reported. Delirium occurred in 25% of patients, while in-hospital and 30-day mortality rates were 3% and 9%, respectively (Table 2).

**Table 2:** Clinical outcomes

Outcomes	*N* = 289
HLOS	7 [5–10] (1–49)
Infectious complications	52 (18)
* Surgical wound infection*	7 (2)
* Urinary tract infection*	16 (6)
* Pneumonia*	19 (7)
* Sepsis*	4 (1)
* Other*	6 (2)
Delirium	72 (25)
In hospital mortality	9 (3)
30-day mortality	26 (9)

Data depicted as median [IQR] (min–max), or as absolute count (percentage), *HLOS* = hospital length of stay

### Frailty parameters

Frailty parameters, including high ASA score, CCI, residing in a nursing home, New Mobility Score (NMS), BMI and albumin on admission, were evaluated in relation to neutrophil phenotype categories (Table 3). These parameters did not differ significantly between the categories. Notably, patients in category 2b included a higher proportion of nursing home residents, although this observation was not statistically significant.

**Table 3:**
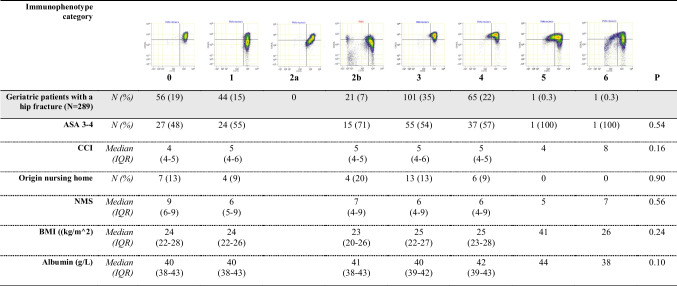
Frailty parameters in relation to immunophenotype categories

The second, grey highlighted row, displays percentages of the entire group. The non-highlighted rows, display the percentage of the total amount of geriatric patients with a hip fracture in that column, or the median with IQR. The p values of Kruskal–Wallis H test displayed. Significant p values indicated with *. *CCI* = Charlson Comorbidity Score. *NMS* = New Mobility Score. *BMI* = body mass index

### Neutrophil categories

The distribution of neutrophil categories in age matched adults without a hip fracture (median age 72) and geriatric patients with a hip fracture (median age 82) showed distinct patterns, as depicted in immunophenotype categories 0 through 6. Among age matched adults without a hip fracture (*N* = 45), the majority (69%) were classified into category 0, with smaller numbers in categories 1 (22%) and 3 (9%). None were observed in categories 2, 4, 5, or 6. In contrast, patients with a hip fracture (*N* = 289) demonstrated more variability across categories, with the largest group in category 3 (35%), followed by categories 4 (22%), 0 (19%), and 1 (15%). Age matched controls were significantly more likely to be classified in category 0 compared to patients with a hip fracture (*p* < 0.001).

A neutrophil phenotype not previously observed was characterized by distinctive characteristics. Unlike other categories that typically display two additional distinct subsets, one CD16^low^ and one CD62L^low^, this category showed a diagonal pattern from the lower right to the upper left, indicating a different mixture of subsets. This phenotype is also characterized by a higher proportion of CD62L^low^ cells, suggesting increased activation or priming. As shown in Table 4, geriatric patients with a hip fracture in this category (2b) presented with significantly elevated leukocyte counts (median 11.9) and CRP levels (median 43). Although not statistically significant, patients in this category also demonstrated a trend toward higher rates of infectious complications (24%) and increased 30-day mortality (19%). Patients in immunophenotype category 6 experienced significantly more severe infectious complications compared to other categories (*p* < 0.01).

**Table 4:**
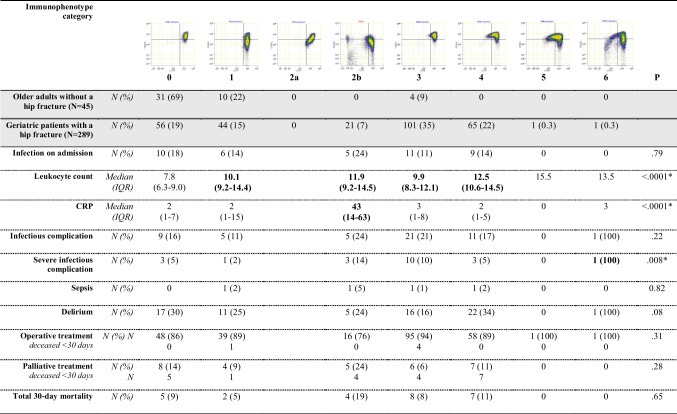
Clinical characteristics and outcome variables of geriatric patients with a hip fracture in different immunophenotype categories and compared to age matched controls

The second and third, grey highlighted rows, display percentages of the entire group. The non-highlighted rows, display the percentage of the total amount of geriatric patients with a hip fracture in that column, or the median with IQR. The p values of Kruskal–Wallis H test displayed. Significant p values indicated with *. Categories with significant differences compared to Category 0 after post hoc testing indicated with bold. *CRP* = C-reactive protein

In the group of patients receiving palliative care, mortality rates within 30 days varied markedly across neutrophil phenotype categories. Among patients classified in categories 0 and 1, 50% died within 30 days, whereas in higher categories (2b, 3, and 4), mortality increased to 83% (Table 4).

## Discussion

This study aimed to examine the association between neutrophil categories and clinical outcomes in geriatric patients with hip fractures, utilizing fully automated flow cytometry, which can be used 24/7 in the central laboratory or as part of a PoC approach. The study included age matched controls, providing unique insights into baseline immune function of the ageing population. The findings of this research provide valuable information on the potential role of neutrophil categories in guiding treatment decisions for this frail population.

The distribution of neutrophil categories in geriatric patients with a hip fracture differed significantly from age matched controls. While the majority of healthy older volunteers (69%) exhibited a typical, healthy neutrophil phenotype (category 0), a broader spectrum of categories was observed in patients with a hip fracture. A substantial proportion of patients (35%) exhibited a category 3 phenotype, indicative of moderate immune activation, while other categories characterized by more pronounced systemic inflammation and neutrophil dysfunction (categories 4 and 6) were also present. This indicates that the occurrence of category 4–6 is triggered by tissue damage caused by the hip fracture and cannot be attributed to aging alone.

Particularly noteworthy was the finding of a novel phenotype, classified as category 2b and characterized by a mixed diagonal distribution of CD16^low^ and CD62L^low^ subsets that seems particularly present in older individuals. This category is the mirror image of the previously described category 2, which predominantly occurs in younger individuals [19]. Therefore, it was chosen to designate the previously known category 2 as 2a and the new category as 2b. Patients in category 2b seemed to include a higher proportion of nursing home residents, demonstrated elevated inflammatory markers, including higher leukocyte counts and CRP levels, and showed a trend toward increased infectious complications and 30-day mortality. Although the association between this phenotype and adverse clinical outcomes was not statistically significant, its clinical relevance remains evident. The elevated CD62L^low^ cells in category 2b suggest a systemic inflammatory response that is primed or activated, which may contribute to increased susceptibility to infection, delirium and a worsened prognosis [22, 28]. It is clinically suspected that this phenotype is more common in individuals with a hip fracture who have been lying on the ground for an extended period before being found. However, as this factor was not systematically recorded or incorporated as a parameter in this study, this hypothesis is yet to be confirmed.

Existing frailty parameters such as ASA, CCI, BMI, albumin, and the New Mobility Score are recognized as essential tools for assessing patient health and guiding shared decision-making (SDM) in geriatric care [29]. These measures are integral to a holistic approach, capturing all aspects of patient vulnerability. However, our data showed that these traditional frailty parameters did not correlate with neutrophil phenotype categories. This suggests that immune phenotypes may represent an independent dimension of frailty, offering novel insights that are not captured by static parameters. Building on this observation, measuring in vivo immune functionality could complement traditional models, such as the Nottingham and Almelo Hip Fracture Scores, which rely on static parameters [15, 16].

This study included patients that received palliative NOM. The decision to start palliative treatment was consistently preceded by a SDM process involving the patient, their family, and the trauma surgeon. Treatment options were carefully weighed within the clinical context and time for reflection was provided to facilitate informed decision-making. These conversations occurred in a calm setting on the ward, though occasionally they took place in the emergency department (ED) if discharge to the patient’s prior care facility was deemed appropriate. In two instances, the anesthesiologist deemed surgery unfeasible due to severe medical contraindications during preoperative evaluation. Regarding previous illnesses and preoperative infections, three-quarters of the patients receiving palliative NOM had cognitive impairments, such as dementia. Other common reasons for opting for palliative care included severe chronic obstructive pulmonary disease (COPD), extensive cardiac disease, advanced malignancies, poor preexisting mobility, and cachexia. Many patients presented with a combination of these comorbidities. While ten patients had an infection upon admission, this was never the sole reason for choosing palliative treatment. For all patients who received palliative treatment, the Almelo Hip Fracture Score (AHFS) was calculated. Among patients with a high AHFS (high risk of early mortality) and a low neutrophil category (0–1), 50% died within 30 days. These findings suggest that neutrophil categories provide additional prognostic value beyond the AHFS alone. Collectively, these results highlight the multifactorial nature of decision-making in this vulnerable population.

Thus, an important contribution of this study is the potential utility of differences in distribution of neutrophil categories as objective biomarkers to support SDM in geriatric patients with a hip fracture. This is especially relevant given the challenges associated with SDM in the acute trauma setting, where treatment decisions regarding operative management versus non operative management are often made with a degree of uncertainty regarding outcome, and without comprehensive advance care planning [30]. Specifically, patients receiving palliative care exhibited a significant variation in 30-day mortality between patients with different neutrophil categories. Mortality rates in patients with higher neutrophil categories (2b, 3, and 4) were considerably higher compared to those with less inflammation (categories 0 and 1). Although the statistical significance for this association was borderline (*p* = 0.05), the potential clinical implications are substantial. In palliative care, where the focus is not to restore mobility or independence, but on pain management and quality of life and quality of dying. Identifying patients at higher risk for early mortality could possibly inform more tailored treatment approaches [12]. Still, it is critical to examine the quality of life in palliative care patients who survive beyond 30 days, as these individuals may have different care needs compared to those who experience early mortality. Moreover, while our findings highlight the potential utility of neutrophil categories in clinical decision making, this remains an exploratory study. For example, patients in category 0–1 still had a 30% risk of delirium, a severe complication with potential long-term consequences [31]. Further longitudinal and interventional studies are required to before integration of neutrophil phenotyping in routine clinical practice.

In conclusion, our data show that it is easily feasible to determine the state of the neutrophil driven innate immune response in geriatric patients with a hip fracture. Furthermore, the identification of different neutrophil categories, as read-out for systemic inflammation could potentially support the shared decision-making process concerning palliative care. Patients in category 0–1 could be deemed fit for surgery, when other risk factors are absent. However, further research should be conducted into the quality of life of these patients that were still alive after 30 days, and into longitudinal immune surveillance during hospitalization, surgery and treatment. Understanding these factors is crucial for optimizing care and guiding treatment decisions in geriatric trauma patients.

## Supplementary Information

Below is the link to the electronic supplementary material.

Supplementary file1 (PDF 201 KB)

## Data Availability

No datasets were generated or analysed during the current study.
