# Supplementary material for: Exploring the role of systemic inflammation in guiding clinical decision making for geriatric patients with a hip fracture
Source: Eur J Trauma Emerg Surg. 2025 May 6;51(1):192. doi: 10.1007/s00068-025-02875-x (PMC12053202; doi:10.1007/s00068-025-02875-x)
Supplement: Supplementary file 1 — Supplementary file1 (PDF 201 KB) [file 68_2025_2875_MOESM1_ESM.pdf]

Supplementary Information

Article title: Exploring the role of systemic inflammation in guiding clinical decision making for geriatric patients with a hip fractureAuthor names: E.J. de Fraiture, T.M.P Nijdam, F.J.C. van Eerten, H.J. Schuijt, A. Bikker, L. Koenderman, F. Hietbrink, D. van der Velde
Corresponding author: E.J. de Fraiture, Department of Trauma Surgery, University Medical Center Utrecht, The Netherlands. E-mail address: e.j.defraiture-3@umcutrecht.nl

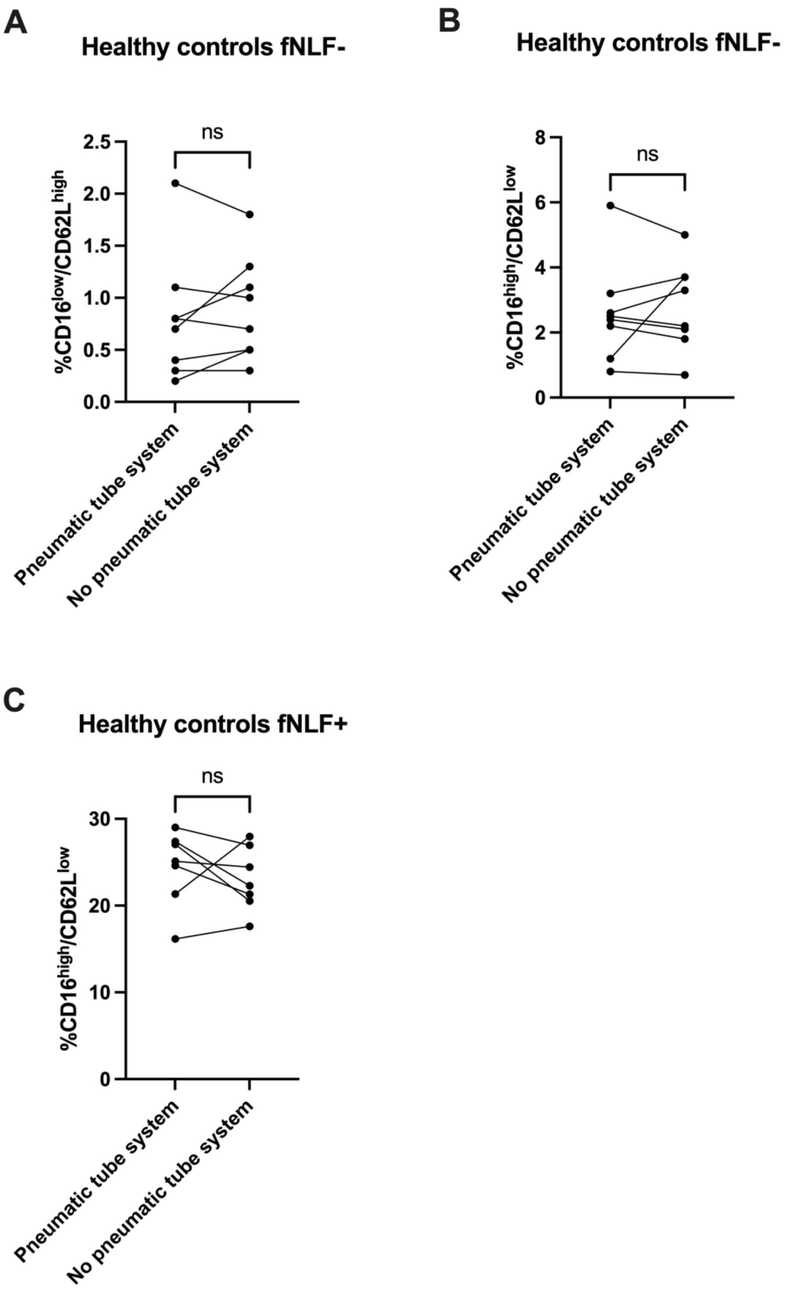

Supplementary Fig. 1 Effect of pneumatic tube transport on neutrophil subsets in healthy control blood samples.
Panel A shows the percentage of $CD16^{low}CD62L^{high}$ neutrophils without fNLF stimulation, Panel B shows the percentage of $CD16^{high}CD62L^{low}$ neutrophils without fNLF stimulation, and Panel C shows the percentage of $CD16^{high}CD62L^{low}$ neutrophils with fNLF stimulation. Duplicate measurements were performed on samples transported via pneumatic tube and those transported manually. Wilcoxon rank test revealed no significant differences between the conditions.
